# 3D‐Printed Radiopaque Microdevices with Enhanced Mucoadhesive Geometry for Oral Drug Delivery

**DOI:** 10.1002/adhm.202201897

**Published:** 2022-12-04

**Authors:** Tien‐Jen Chang, Rolf Bech Kjeldsen, Juliane Fjelrad Christfort, Eduard Marzo Vila, Tommy Sonne Alstrøm, Kinga Zór, En‐Te Hwu, Line Hagner Nielsen, Anja Boisen

**Affiliations:** ^1^ The Danish National Research Foundation and Villum Foundation's Center for Intelligent Drug Delivery and Sensing Using Microcontainers and Nanomechanics (IDUN) Department of Health Technology Technical University of Denmark Kgs. Lyngby 2800 Denmark; ^2^ Department of Applied Mathematics and Computer Science Technical University of Denmark Kgs. Lyngby 2800 Denmark; ^3^ BioInnovation Institute Foundation Copenhagen 2200 Denmark

**Keywords:** gastrointestinal tracking, microcontainers, microscale 3D printing, mucoadhesion, stereolithography, X‐ray imaging

## Abstract

During the past decades, microdevices have been evaluated as a means to overcome challenges within oral drug delivery, thus improving bioavailability. Fabrication of microdevices is often limited to planar or simple 3D designs. Therefore, this work explores how microscale stereolithography 3D printing can be used to fabricate radiopaque microcontainers with enhanced mucoadhesive geometries, which can enhance bioavailability by increasing gastrointestinal retention. Ex vivo force measurements suggest increased mucoadhesion of microcontainers with adhering features, such as pillars and arrows, compared to a neutral design. In vivo studies, utilizing planar X‐ray imaging, show the time‐dependent gastrointestinal location of microcontainers, whereas computed tomography scanning and cryogenic scanning electron microscopy reveal information about their spatial dynamics and mucosal interactions, respectively. For the first time, the effect of 3D microdevice modifications on gastrointestinal retention is traced in vivo, and the applied methods provide a much‐needed approach for investigating the impact of device design on gastrointestinal retention.

## Graphical abstract

For the first time, this study demonstrates how an innovative high definition digital versatile disc based microscale 3D printing setup can be used to fabricate versatile microdevices, with adhering geometric structures and incorporated contrast agent, for oral drug delivery. Generally, the presented fabrication process improves mucoadhesion and traceability of microdevices used for oral drug delivery.

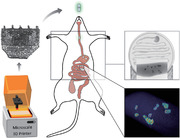

## 1 Introduction

Most patients prefer the oral administration route for their intake of drugs due to convenience that results in a high patient compliance.^[^ ^1^ ^]^ Additionally, oral dosage forms do not require sterile production or trained personnel for administration, which all together lowers their production cost compared to, for example, injectables.^[^ ^2^ ^]^ However, several challenges related to the gastrointestinal (GI) tract must be addressed before the delivered drug can be successfully absorbed from the intestine. These include a steep pH gradient and several digestive agents, such as bile salts, enzymes, and pancreatic secretions. Furthermore, the inner surface of the GI tract is covered by a mucus layer of varying thicknesses, which is the last line of defense for the body to avoid absorption of unwanted compounds.^[^ ^3^ ^]^

During the past decades, several microfabricated drug delivery devices have been developed to address the challenges within oral drug delivery. Despite significant design differences, most of them are based on the same overall structure, namely a central reservoir for drug loading offering protection until the dissolution of a polymeric lid triggers a unidirectional drug release. However, it has been shown that specific changes in the design can improve the chance for successful oral delivery of drugs. For example, planar poly(methyl methacrylate) microdevices have shown significantly improved retention time in the small intestine, leading to a 4.5‐fold increase for the bioavailability of acyclovir in mice after oral dosing.^[^ ^4^ ^]^ Recently, therapeutical devices with sharp microtips, referred to as theragrippers, have been fabricated. They spontaneously fold at the temperature of the GI tract with a force sufficient to penetrate the mucus layer, which resulted in colonic retention in living rats for 24 h after rectal administration.^[^ ^5^ ^]^

While planar microdevices have been highlighted for their resistance to shear stress, microdevices with a larger aspect ratio, such as microcontainers, are beneficial considering their large loading capacity and potential embedment in the mucus layer.^[^ ^6^ ^]^ Microcontainers are polymeric devices (typically 200–300 µm in size) with an inner cavity for drug loading and an open topside providing a unidirectional release. They have shown to be advantageous for oral delivery of multiple compounds, including small‐molecule drugs,^[^ ^7^, ^8^, ^9^ ^]^ peptides,^[^ ^10^, ^11^ ^]^ probiotics,^[^ ^12^ ^]^ and vaccines.^[^ ^13^ ^]^ Additionally, from an in situ perfusion model in rats, microcontainers have been shown to possess inert mucoadhesive properties.^[^ ^8^ ^]^ The mucoadhesive polymers chitosan and polyethylene glycol (PEG) have been studied for chemical functionalization of the open topside to increase mucoadhesion.^[^ ^10^ ^]^ However, the improved mucoadhesion observed ex vivo has not translated into significant alterations in drug absorption in vivo.^[^ ^14^, ^15^ ^]^ Most recently, physically functionalized cubic microcontainers were found to adhere significantly better to the mucosa than cylindrical microcontainers after in situ perfusion in the rat colon.^[^ ^16^ ^]^

A commonly used manufacturing technique is 3D printing, which allows for more advanced geometry designs and higher fabrication flexibility than conventional methods.^[^ ^17^, ^18^ ^]^ It offers a substantial advantage when creating innovative geometries and physical functionalizations for drug delivery devices in order to achieve, for example, controlled release or co‐delivery.^[^ ^19^, ^20^, ^21^ ^]^ In addition, the simplicity of the 3D printing process can reduce the fabrication time of different dosage forms and therefore, 3D printing is recognized as a revolutionary approach for realizing mass customization of personalized drugs.^[^ ^22^ ^]^ Earlier, a millimeter‐sized drug carrier with anchor‐like features has been designed and fabricated, using a digital light processing 3D printer with a printing resolution of tens of micrometers, to expand the contact area with intestinal mucus.^[^ ^23^ ^]^ These anchor‐like features showed extended retention time in an ex vivo model. However, due to limitations in printing resolution, 3D‐printed oral drug devices are fabricated at the centimeter to millimeter scale, and they are challenging to miniaturize.^[^ ^20^ ^]^ A newly developed two‐photon technique demonstrated the opportunity to 3D print with nanoscale resolution that can be used to achieve complex structures on the surface of microdevices.^[^ ^17^ ^]^ However, this two‐photon technique has several limitations, such as microscale printing volume and slow printing speed, resulting in a low throughput.^[^ ^24^, ^25^, ^26^ ^]^ Furthermore, its high cost leads to low economic benefit for the pharmaceutical industry. Recently, our custom‐built high definition digital versatile disc (HD‐DVD) based 3D printer achieved micro‐ and nanoscale printing resolution.^[^ ^27^ ^]^ This 3D printer is cost‐effective and without limitations in the available print volume size. For this reason, it is a promising alternative for scaled‐up fabrication of microdevices with sizes ranging from tens to hundreds of micrometers. Moreover, the method can create structures with a size of tens of micrometers (close to the dimension of small intestinal villi) on the surface of the microdevices to enhance their mucoadhesion. Despite the promising potential for microdevices in oral drug delivery, the mucoadhesive properties are still challenging to assess in vivo. Due to a generally frequent discrepancy between in vitro and in vivo observations within oral drug delivery,^[^ ^28^, ^29^ ^]^ there is an urgent need for improved imaging techniques to locate and track microdevices in the GI tract. Different imaging techniques have been suggested for in vivo GI tracking,^[^ ^30^ ^]^ however most of them have so far not facilitated fast and easy imaging of individual 3D microdevice structures. Previously, microdevices (more specifically microcontainers) have been loaded with an X‐ray contrast agent, barium sulfate (BaSO_4_), resembling a drug carrier.^[^ ^31^ ^]^ However, in this way, the structural device itself is not imaged, and differences in 3D design and the impact of this cannot be clearly visualized.

X‐ray imaging, composed of planar X‐ray imaging and computed tomography (CT) scanning, is being widely used for different medical applications, ranging from conventional procedures to advanced (pre‐)clinical studies on passive and active targeted diagnosis of tumors^[^ ^32^ ^]^ and 3D bioprinting of artificial organs and tissues for surgical purposes.^[^ ^33^ ^]^ Furthermore, X‐ray imaging for GI tracking of orally dosed capsular devices in the millimeter scale has previously been carried out to better understand their GI behavior in rats,^[^ ^34^, ^35^ ^]^ dogs,^[^ ^36^ ^]^ and humans.^[^ ^37^ ^]^ In fact, X‐ray imaging has been utilized for our research within quantitative GI tracking of microscale oral drug delivery devices in rats.^[^ ^31^ ^]^ However, the loading of an X‐ray contrast agent, for example BaSO_4_, was required to make them traceable with X‐rays in the GI tract. In a recent study with microcontainers, BaSO_4_ occupied all the void space and left no room for loading of a potential drug. Therefore, it is of great interest to enable tracking of the devices with delivery of drugs by incorporating BaSO_4_ into the shell of the microcontainers. Previously, a similar approach has been used to prepare radiopaque microspheres by utilizing thermally induced phase separation^[^ ^38^ ^]^ and a one‐step electrospraying method,^[^ ^39^ ^]^ respectively, that were successfully tracked in vivo with X‐ray imaging.

**Figure 1:**
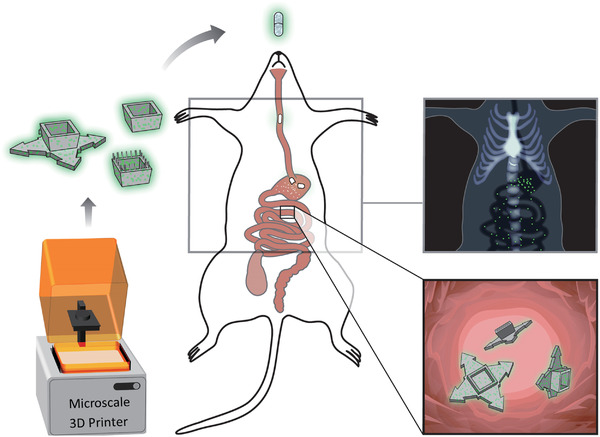
Schematic illustration of the experimental setup. Radiopaque microcontainers were 3D‐printed with three different designs (utilizing microscale 3D printing) and subsequently dosed to rats in gelatin capsules using oral gavage. Their location in the GI tract was determined by planar X‐ray imaging 0.5–3 h after dosing. Further investigation of spatial dynamics and mucosal interactions in the small intestine was carried out using CT scanning and CryoSEM, respectively.

Here, for the first time, an approach for 3D printing and evaluating radiopaque microdevices for oral drug delivery with enhanced mucoadhesive geometries is presented (**Figure** **1**). Some of the geometries included are open inverted 3D trapezoid structures with additional features such as pillars and arrows, which are believed to increase mucoadhesion due to the possibility for an increased entanglement.^[^ ^5^, ^16^, ^23^ ^]^ The mucoadhesive properties of the microcontainers are evaluated ex vivo by high‐precision cantilever force measurements and in vivo in rats using X‐ray imaging after oral dosing. In addition to quantifying the GI retention and transit time using planar X‐ray imaging, further investigation of spatial dynamics and mucosal interactions in the small intestine is made using CT scanning and cryogenic (Cryo) scanning electron microscopy (SEM), respectively.

## 2 Results and Discussion

### 2.1 Fabrication of 3D‐Printed Microcontainers

**Figure 2:**
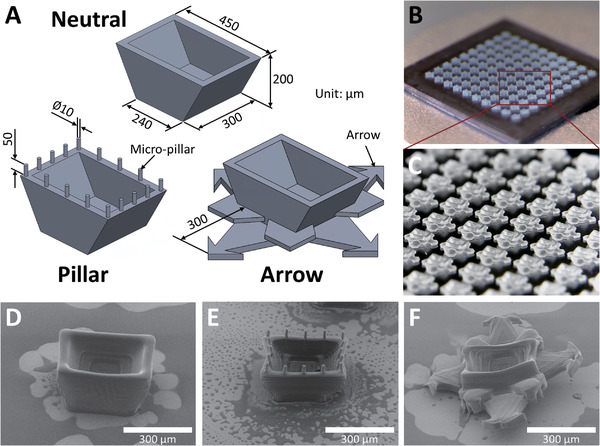
3D‐printed biocompatible microcontainers with enhanced mucoadhesive geometry. A) Illustration of neutral, pillar, and arrow microcontainer design. B) Picture of 3D‐printed microcontainers in a 10 × 10 matrix on a silicon chip. C) Detailed microscope image of 3D‐printed microcontainers. SEM images of D) neutral, E) pillar, and F) arrow microcontainers (Figure S1A–C, Supporting Information, for mass‐production).

Three microcontainer designs were created and fabricated trying to control the orientation of unidirectional drug release while extending the GI retention and transit time. Considering the promising mucoadhesion of cubic microcontainers in situ, previously reported,^[^ ^16^ ^]^ all microcontainer designs were based on square‐like shapes. First, microcontainers with a large contact area on the top side (with a cavity) and a small contact area at the bottom side were 3D‐printed (neutral design, **Figure** **2**A). This design appeared as an open inverted truncated pyramid structure, which might increase the probability of having a unidirectional drug release toward the mucosa. The dimension of these neutral microcontainers were 450 × 300 × 200 µm^3^ (length × width × height), while the length of the bottom side was 240 µm. For the second design, 16 micropillars with a diameter and height of 10 and 50 µm, respectively, were printed on the top side of neutral microcontainers (pillar design, Figure 2A). These micropillars were hypothesized to penetrate the mucin network and thus to enhance mucoadhesion. Finally, microcontainers were designed with four arrow structures, each 300 µm long, and four minor spiky plates on the side to drastically increase the surface area (arrow design, Figure 2A). The arrow design was expected to hook into the mucus layer for a deeper entanglement, thereby extending the retention and transit time.

The three design concepts were realized using the custom‐built micro‐ and nanoscale 3D printer.^[^ ^27^ ^]^ The 3D printer cured the biocompatible photopolymer to produce 100 microcontainers in a 10 × 10 matrix on a silicon chip (Figure 2B,C). The neutral microcontainers were fabricated with an inner cavity for drug loading, which appeared clean and without residual photopolymer in the cavities after a washing procedure (Figure 2D). The sixteen micropillars were printed onto the wall of the microcontainers (Figure 2E), whereas the arrow structures were printed onto the bottom side of the microcontainers (Figure 2F). All three designs were successfully mass‐produced by 3D printing showing high homogeneity (Figure S1A–C, Supporting Information).

The utilized 3D printer enabled scaled up fabrication of microcontainers with microscale features on a relatively large area (10 × 10 mm^2^), which would be challenging to achieve with other 3D printing methods.^[^ ^27^ ^]^ For the neutral design, a clear overhang asymmetrical structure was achieved. Even with a small contact area at the bottom, the microcontainers still adhered well to the substrate. Thus, the subsequent drug loading and lid coating processes could easily be implemented, while keeping the devices fixed on the substrate. The micropillars added to the pillar design were successfully 3D‐printed with the dimensions of 11.5 × 47.4 µm^2^ (diameter × height) and were thereby sufficiently small to penetrate the mucin network comprising numerous tens‐of‐micrometers holes.^[^ ^40^ ^]^ A single print batch took ≈12 h and thus, the manufacturing capacity was suitable for producing microcontainers for an in vivo study. The printing time can be further optimized by tuning printing speed and laser intensity.

The printing parameters were tuned and optimized for each microcontainer design trying to thoroughly compensate for limitations of the laser, which might cause minor dimension variations. For the arrow design, it was challenging to obtain a sharp tip on the arrows due to the accumulation of relatively more laser energy close to the tips where the motor of the 3D printer stage slowed down. Additionally, the arrow structures bent slightly away from the substrate after the washing process, which is believed to be caused by accumulation of inner stress during the 3D printing process. This slight deformation was not expected to affect the overall functionality. In fact, the lower surface contact might make substrate detachment easier during the later device collecting process.

### 2.2 A Microdevice for Controlled Oral Drug Delivery—In Vitro Proof‐of‐Concept

The 3D‐printed microcontainers were loaded with a small molecule drug, furosemide, (Figure S2A, Supporting Information) and coated with a pH‐sensitive polymer, Eudragit L100 (Figure S2B, Supporting Information) to evaluate if they were suitable as drug carriers for oral delivery. Furosemide was used as a model drug because previous studies have shown microcontainers to increase the bioavailability of this drug by 220% compared to using standard gelatin capsules.^[^ ^8^ ^]^ The purpose of the present study was not to investigate pharmacokinetics in great details but to proof that the 3D‐printed microcontainers have the potential for being used for oral drug delivery in a similar way as for previously reported types of microcontainers. An Eudragit L100 coating was chosen as it is typically used for oral drug delivery to the small intestine due to its suitable pH sensitivity, and because it is not affected by enzymatic activity.

The release of furosemide was measured in media simulating gastric and intestinal pH conditions, respectively (Figure S2C, Supporting Information). Each substrate chip, holding 100 microcontainers, was loaded with 1.0 ± 0.2 mg furosemide, corresponding to ≈10 µg per microcontainer. This was a substantially larger loading capacity than previously reported for similarly sized microdevices, which was 1.5^[^ ^4^ ^]^ and 3–5 µg per microdevice,^[^ ^41^ ^]^ respectively. This higher loading capacity can be achieved since the structural sidewalls of the microdevices are thinner when fabricated by the custom‐built 3D printer.^[^ ^27^ ^]^

An in vitro release profile for the furosemide‐loaded microcontainers was obtained using a µDISS Profiler. This is a commonly applied method for in vitro evaluation of drug release from microcontainers before animal studies.^[^ ^7^, ^8^, ^9^ ^]^ The release was studied in a two‐step model simulating the pH in the GI tract of fasted rats, where a 30 min gastric step (4 mm hydrochloric acid pH 2.4) was followed by an intestinal step (phosphate buffered saline (PBS), pH 7.5) until a complete release was achieved. During the gastric step, only 4.4 ± 1.0% (mean ± standard deviation (SD), *n* = 4) of the loaded furosemide was released, and after changing to the intestinal medium, furosemide was gradually released until 90 min after initiating the study. This release profile was consistent with the release observed from microcontainers fabricated by other methods.^[^ ^7^, ^9^ ^]^ Thus, when combined with a pH‐sensitive polymeric coating, the 3D‐printed microcontainers showed promising results as an oral drug delivery device for targeted delivery to the small intestine.

### 2.3 Ex Vivo Mucoadhesion Force Measurements

**Figure 3:**
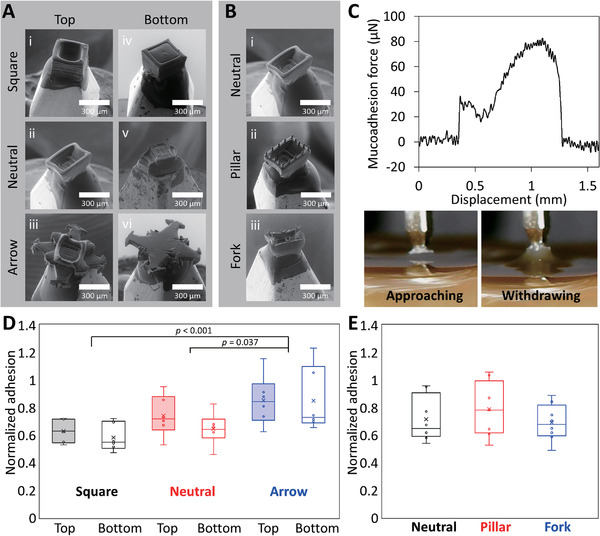
Ex vivo mucoadhesion characterization of 3D‐printed microcontainer. A) SEM images of the top side of a i) square, ii) neutral, or iii) arrow microcontainer on the microprobe, and the bottom side of a iv) square, v) neutral, or vi) arrow microcontainer on the microprobe. B) SEM images of the top side of a i) neutral, ii) pillar, or iii) fork microcontainer on the microprobe. C) Mucoadhesive force versus displacement curve while approaching and withdrawing bottom side of an arrow microcontainer to porcine small intestinal tissue. D) Orientation study with normalized mucoadhesion of square, neutral, and arrow microcontainer's top and bottom sides. Mean ± SD, *n* = 6, Tukey's pairwise test. E) Additive feature study shows normalized mucoadhesion of neutral, pillar, and fork microcontainer's top side. Mean ± SD, *n* = 6.

The wide‐range force analyzer^[^ ^42^ ^]^ was used to measure the mucoadhesive forces of microcontainers in order to predict potential interactions with mucus. Chemical functionalization (i.e., lid coating) using Eudragit L100 was expected to increase ex vivo mucoadhesion but most likely would not affect GI retention and transit, as previously reported for chitosan and polyethylene glycol.^[^ ^14^, ^15^ ^]^ Therefore, to only focus on how the physical functionalization of the 3D‐printed microcontainers would affect mucoadhesion, none of them were coated for the ex vivo force measurements and also not for the later in vivo study when investigating the potential translation into increased retention and transit. Two types of ex vivo studies were implemented to investigate the orientation of microcontainers and the mucoadhesion of the added features, respectively. In both studies, porcine small intestinal tissue was utilized. In the orientation study, the dynamic orientation of microcontainers was predicted by comparing the mucoadhesion of both sides of the devices. As a control, cubic symmetrical microcontainers (square design) in the size of 300 × 200 µm^2^ (width × height) were 3D‐printed and compared with the asymmetrical neutral and arrow designs. The microcontainers were glued onto a microprobe to facilitate contact between mucus and the top/bottom side (**Figure** **3**A,B). The force analyzer continuously recorded the mucoadhesive forces while approaching and withdrawing the microcontainers (Figure 3C). The adhesive force of the microprobe itself was measured for each tissue and used to normalize between measurements.

The obtained results (Figure 3D) indicated that the square microcontainers had the same level of mucoadhesion on both sides. The top side of neutral microcontainers showed a slightly larger mucoadhesion than the bottom side, which was expected due to the asymmetrical design. A two‐way analysis of variance (ANOVA) was carried out to test the effects of both the orientation and the design. The statistical result indicated that the top‐bottom orientation did not have a significant difference for these three designs (*p* = 0.34). Instead, the overall device design showed a significant effect on mucoadhesion (*p* < 0.01). A Shapiro‐Wilk normality test was computed (*p* < 0.05) to confirm that the normality assumption of ANOVA was satisfied.^[^ ^43^ ^]^ A Tukey's honestly significant difference test was used to analyze the individual means further.^[^ ^44^ ^]^ The arrow microcontainers showed a significant enhancement of mucoadhesive forces compared to the square (*p* < 0.001) and neutral (*p* = 0.037) microcontainers. These results confirmed that a larger contact area at the microscale increases mucoadhesion. Moreover, the bottom side of both the neutral and square microcontainers had a similar degree of mucoadhesion, even though the neutral design had a smaller bottom side area. The reason for this might be that the lateral slope area of the bottom side of neutral microcontainers was still in contact with the mucus.

In the study of additive features, the measured mucoadhesive force was compared for the topside of the neutral design to the topside of microcontainers with added features (Figure 3E). From the normalized mucoadhesive force results, it was observed that the micropillars provided a slightly, nonsignificant, enhanced mucoadhesion compared to the neutral design and to microcontainers containing pairs of spikes (fork design), respectively.

### 2.4 Incorporation of BaSO_4_ into 3D‐Printed Microcontainers

**Figure 4:**
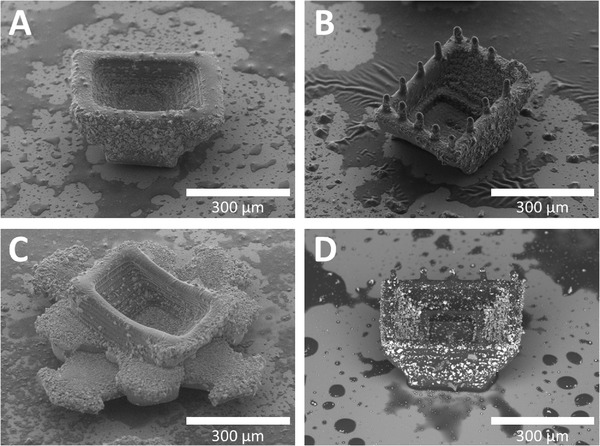
Incorporation of BaSO_4_ into 3D‐printed microcontainers. SEM images of 3D‐printed radiopaque microcontainers with A) neutral, B) pillar, and C) arrow design, respectively (Figure S1D–F, Supporting Information, for mass‐production). D) High contrast SEM image clearly indicating the BaSO_4_ particles in the pillar design.

For being able to trace the 3D‐printed microcontainers throughout the entire GI tract, an X‐ray contrast agent of BaSO_4_ nanoparticles was incorporated into their shell (**Figure** **4**A–C and Figure S1D–F, Supporting Information). Prior to 3D printing, the photopolymer was mixed with BaSO_4_ nanoparticles. Neutral, pillar, and arrow microcontainers, 400 units in total, were produced for a proof‐of‐concept animal study. All three designs maintained the same scale and structure as microcontainers printed without BaSO_4_, and detailed SEM analysis of the pillar design revealed a clear contrast provided by the incorporated BaSO_4_ nanoparticles (Figure 4D).

**Figure 5:**
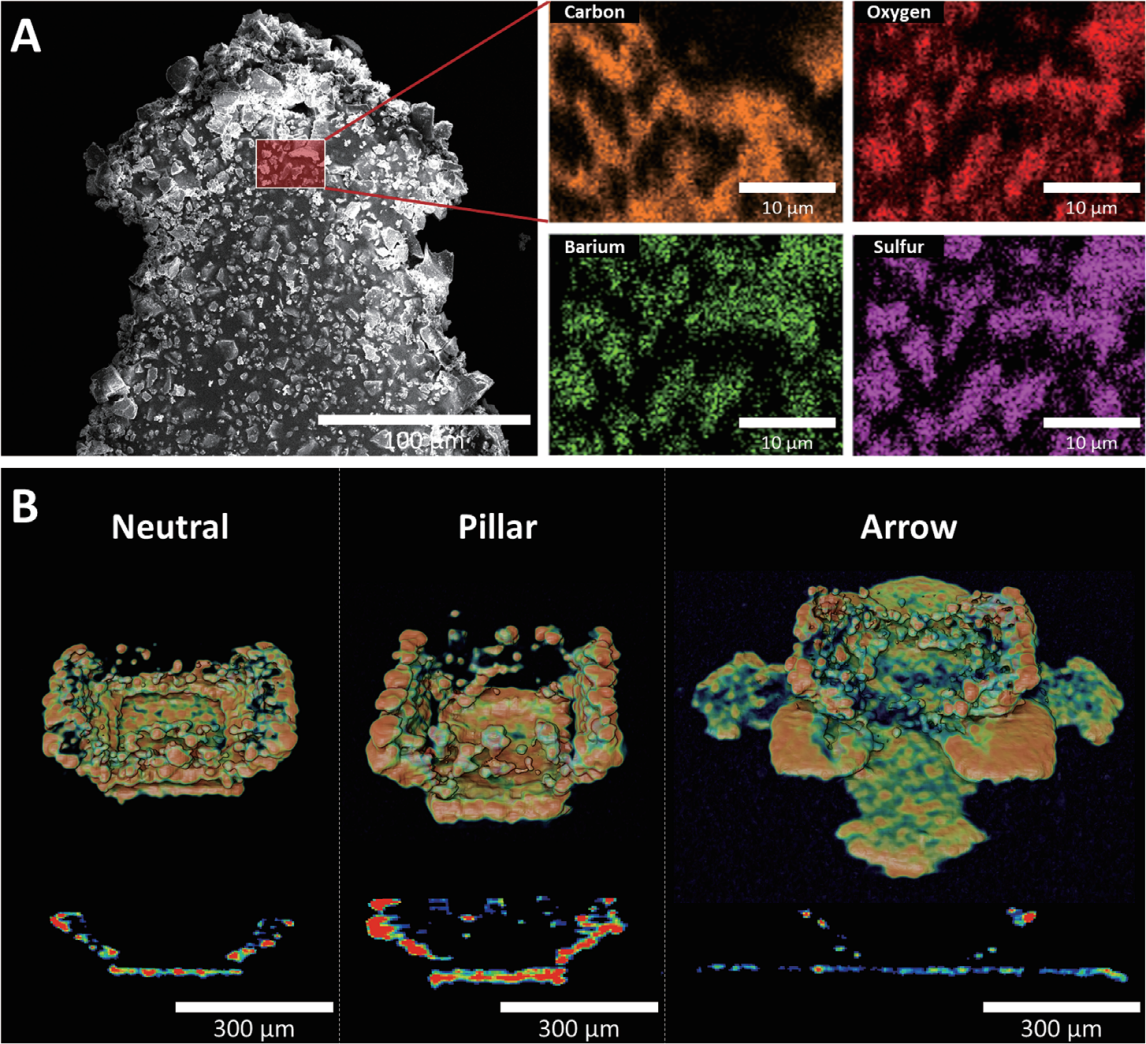
EDX analysis and µCT scanning. A) SEM image of a radiopaque arrow microcontainer's surface and EDX analysis, which includes carbon, oxygen, barium, and sulfur. B) µCT scanning showing the homogeneity of BaSO_4_ nanoparticles in the 3D‐printed radiopaque microcontainers (Figure S3, Supporting Information, for multiple microcontainers).

An energy dispersive X‐ray (EDX) analysis was performed to investigate the surface distribution between the photopolymer and the BaSO_4_ nanoparticles. As expected, carbon, oxygen, barium, and sulfur were detected and mapped at the microcontainer surface (**Figure** **5**A). Additionally, the SEM image revealed a rough surface with numerous protruding microparticles. Furthermore, µCT scans indicated that BaSO_4_ nanoparticles were inhomogeneously distributed in the 3D‐printed structures (Figure 5B and Figure S3, Supporting Information). The reason for this is the general high insolubility of BaSO_4_. During the beginning of the ≈12 h print process, the BaSO_4_ nanoparticles were still well dispersed in the photopolymer. However, they gradually sedimented over time, which led to aggregated microparticles especially found at the bottom part of the printed microcontainers.

### 2.5 Quantitative Gastrointestinal Tracking for Retention and Transit Time

**Figure 6:**
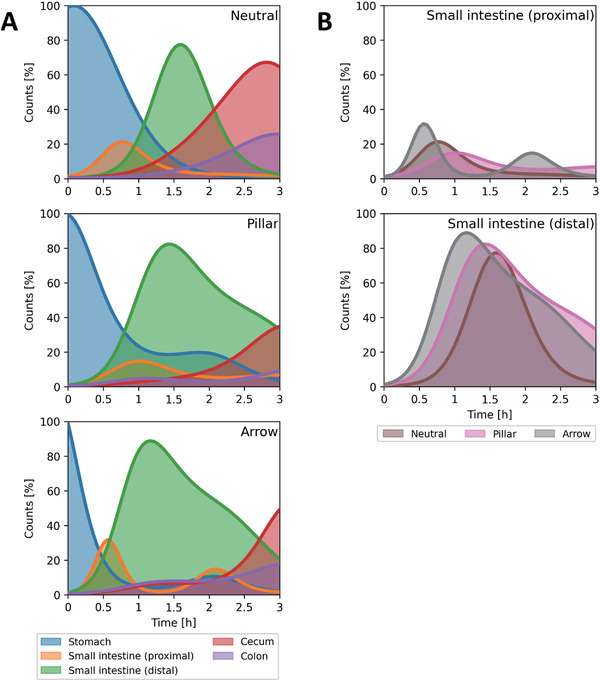
GI retention and transit time of microcontainers. A) Graphs with overlayed fits (from Figure S5, Supporting Information) showing the quantity of microcontainers at specific locations over time for the neutral, pillar, and arrow design, respectively. B) Graphs directly comparing the quantity of microcontainers in the small intestinal sections (proximal and distal) for the three designs.

To evaluate the mucoadhesive properties observed ex vivo for the neutral, pillar, and arrow microcontainer designs, retention and transit times were investigated in an in vivo rat study using planar X‐ray imaging for quantitative GI tracking, similarly to what has previously been demonstrated.^[^ ^15^, ^31^ ^]^ Prior to the study, having *n* = 2 rats was discussed but chosen in order to reduce the total number of animals needed to look for any apparent differences in GI retention and transit between the different microcontainer designs. Furthermore, an appropriate statistical procedure (Gaussian process with a squared exponential kernel and a Poisson likelihood) was going to be applied on the data obtained. After oral administration of microcontainers and subsequent euthanasia at varying time points, planar X‐ray images of removed GI tracts were acquired (Figure S4, Supporting Information). From these planar X‐ray images, the quantity of microcontainers in different GI sections was counted and normalized (Table S1, Supporting Information), then plotted and fitted as a stochastic process over time (Figure S5, Supporting Information). By overlaying the fitting of each GI section for the three designs (**Figure** **6**A), some clear trends on GI retention and transit were observed as later described. Considering that most orally delivered drugs aim to be released and absorbed in the small intestine, it was essential to look for any differences in retention for the microcontainer designs in this area (Figure 6B). Therefore, the fittings for the small intestinal sections (proximal and distal) for all three designs were overlayed for easier comparison.

From the quantitative gastrointestinal tracking results (Figure 6), it was observed that microcontainers with all three designs appeared in the stomach 0.5 h after dosing. For the neutral and pillar design, 60–70% of the microcontainers were found in the stomach after 0.5 h. In addition for these two designs, 5–10% of the microcontainers were found in cecum after 1 h, which increased to an average of 40–70% after 3 h, while the quantity in the previous GI sections had decreased. On the contrary, only around 20% of the arrow designed microcontainers were observed in the stomach after 0.5 h, while ≈40% of them had already moved on to the proximal and distal small intestine, respectively. In general, for the distal part of the small intestine, the microcontainers were clearly seen to enter this region and increase in number with time before moving on to the large intestine. The majority of the neutral (≈50%), pillar (≈60%) and arrow (≈80%) microcontainers were found in the distal small intestine after 2, 2, and 1 h, respectively. After 3 h, the majority of microcontainers for all three designs were located in the cecum, and for the neutral and arrow design, no considerable quantity of microcontainers could be detected in any other region at this time. However, the pillar design resulted in an equal distribution between the distal small intestine and the cecum after 3 h, indicating trends toward enhanced adhesion of the pillar design in the distal small intestine.

Despite some trends indicating improved retention of the microcontainers with pillars in the small intestine, no significantly increased retention could be observed in rats in vivo. In that regard, the enhanced mucoadhesion measured for the arrow design ex vivo (Figure 3D) did not seem to translate into an increased retention time in the GI tract in vivo. A reason for this might be the relatively large size of the arrow microcontainers, which could make them more sensitive to the flow of GI contents like food and water. A similar discrepancy between ex vivo and in vivo adhesion has previously been observed for SU‐8 microcontainers coated with PEG and chitosan.^[^ ^10^, ^14^ ^]^ Here, microcontainers coated with PEG significantly improved the adhesion ex vivo when compared to uncoated microcontainers.^[^ ^10^ ^]^ However, when this was evaluated in vivo after oral dosing to rats, no difference could be observed in the pharmacokinetic profile for paracetamol, which was loaded into the microcontainers.^[^ ^14^ ^]^ This indicates that the PEG and chitosan coatings did not have a pronounced mucoadhesive effect in vivo. Another study showed a similar trend when investigating microcontainers loaded with mesalazin and coated with Eudragit FS100 and tannic acid‐functionalized zein protein, respectively, ex vivo and in vivo.^[^ ^15^ ^]^ Here, ex vivo mucoadhesive measurements showed a clear variation for the two coatings, but in vivo retention and transit time did not show significant differences. Furthermore, the impact of morphology and geometry on mucoadhesion has been investigated extensively for micro‐ and millimeter‐sized devices in vitro, ex vivo and in situ.^[^ ^16^, ^23^, ^45^, ^46^, ^47^ ^]^ The addition of nano‐ and microsized surface structures was found to improve mucoadhesion in vitro and ex vivo, respectively, while shape has been shown to affect retention in perfusion models ex vivo and in situ.^[^ ^16^, ^46^ ^]^ Although these design modifications remain to be investigated in vivo, the results from the present study indicated that more drastic design changes will be required in order to see a considerable effect in vivo.

In a living animal, peristaltic forces and the flow of foods and liquids represent a challenge that is not simulated properly in vitro and ex vivo. Since in situ studies are performed in live anesthetized animals, they can be utilized to study mucoadhesion in the presence of peristaltic forces. However, the GI transit time has been shown to be reduced by ≈50% in anesthetized rats.^[^ ^48^ ^]^ Recently, active microdevices, such as theragrippers, have demonstrated promising mucoadhesion in vivo.^[^ ^5^ ^]^ Theragrippers spontaneously fold at body temperature with a force sufficient to penetrate the colonic mucus layer with their microtips, and they showed the ability to reside inside the colon of live animals for up to 24 h after rectal administration. In addition, micromotors, capable of propelling themselves into the GI mucus layer, have resulted in improved intestinal retention 6 h after oral dosing, compared to static microparticles.^[^ ^49^ ^]^

### 2.6 Spatial Dynamics and Mucosal Interactions

Mucoadhesive properties of oral drug delivery devices in vivo are not always easily predictable and, as in this case with the present 3D‐printed microcontainers, they might not be straightforward to investigate.^[^ ^50^, ^51^ ^]^ To reach a level deeper than quantitative GI tracking, CT scanning and CryoSEM were used to study the spatial dynamics and mucosal interactions of the microcontainers in the small intestine (Figure S6, Supporting Information). Here, spatial dynamics cover the specific location and overall orientation of the microcontainers, whereas mucosal interactions describe the embedment of microcontainers in the mucus layer and their specific orientation in that regard.

**Figure 7:**
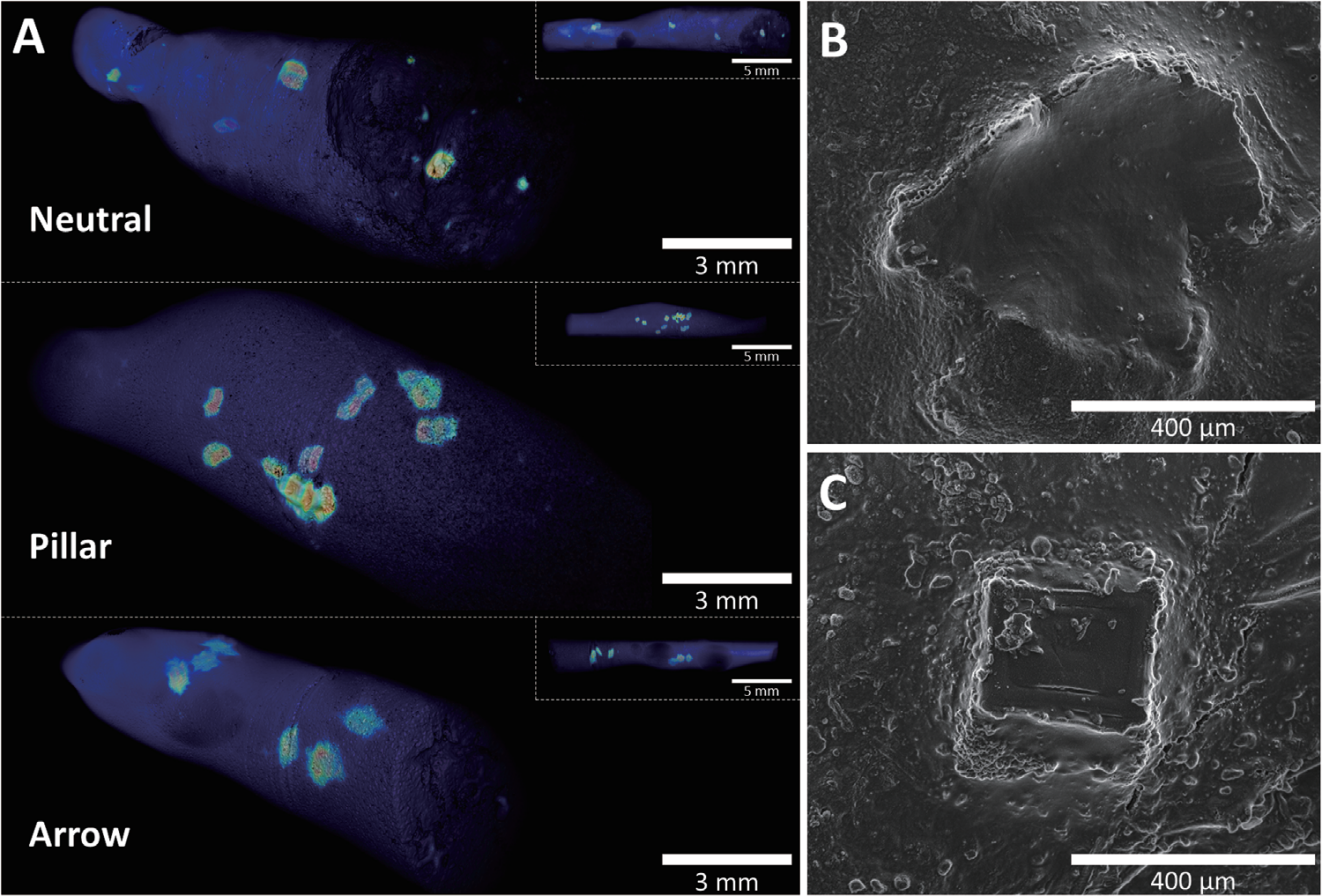
Spatial dynamics and mucosal interactions of microcontainers. A) CT scan images of neutral, pillar, and arrow microcontainers inside small intestinal pieces, revealing their spatial dynamics. Accompanying CT scans movies are found online (Movies S1–S3, Supporting Information). B,C) CryoSEM images of pillar microcontainers showing their mucosal interactions, such as embedment into the intestinal tissue and the microcontainer orientation.

The CT‐scanned intestinal pieces (**Figure** **7**A and Movies S1–S3, Supporting Information) revealed details about the spatial dynamics. For example, it showed the exact location and distribution of the microcontainers but also revealed that they had no preferred orientation toward the intestinal wall. The CryoSEM provided a close observation of the interaction between a microcontainer and the mucus layer. Two microcontainers with pillars were found on the mucus layer in a side‐way posture (Figure 7B) and one was found facing down with the top side in contact with the mucus layer (Figure 7C). These microcontainers were covered by mucus and therefore, only the shape of the microcontainers could be recognized. Further details about the microcontainer surface topography and micropillar features were difficult to see due to this mucus coverage. Furthermore, to investigate any potential intestinal inflammatory response due to the mucosal interaction of the 3D‐printed microcontainers, pathological staining could be applied. This was omitted based on a previously reported finding showing that a deep‐mucus‐penetrating microneedle patch system did not show any of such intestinal inflammatory responses when investigated using hematoxylin and eosin staining and immunofluorescence staining of interleukin‐6.^[^ ^52^ ^]^

### 2.7 3D‐Printed Radiopaque Microcontainers for Oral Drug Delivery

Conclusively, the presented 3D printing process enables microscale fabrication of versatile innovatively structured microdevices for oral drug delivery with an X‐ray contrast agent incorporated into the device itself, which has not been done before. In the future, this will allow for qualitative and quantitative GI tracking using X‐ray imaging, while systemic drug absorption can be simultaneously monitored. Three different microcontainer designs were fabricated and tested, but in the future, more designs with other exceptional adhesive features, such as hooks or branched spikes, can easily be adopted and 3D‐printed. Based on the results from the in vivo rat study, some sort of active or shape changing mechanism seems to be necessary in order to significantly improve the retention and transit time of microdevices in the presence of peristaltic forces and food contents. A few examples of recently reviewed physically interacting modes are injection, jetting, iontophoresis, ultrasound, hyperthermia, magnetism and convection that all disrupt or perturb the epithelium,^[^ ^53^ ^]^ and current work is trying to combine such physical modes with 3D‐printed radiopaque microcontainers. Furthermore, GI retention and transit time in rodents, as concerned in this work, are drastically different than in humans, and especially gastric emptying in rodents occurs faster than in humans. For example, gastric emptying typically proceeds in humans linearly whereas an exponential decay is present in rodents.^[^ ^54^ ^]^ This can be attributed to the difference in feeding patterns, where humans usually achieve nearly complete gastric emptying. For rodents, very short delays between successive food intakes instead result in successive dilution of the stomach contents. For better understanding the GI behavior of 3D‐printed microcontainers in terms of potential human translation, it would be highly relevant to investigate them in large animals, such as rabbits, pigs, and maybe even dogs.

## 3 Experimental Section

### Materials

Silicon wafers (4in, b100N, n‐type) were acquired from Okmetic (Vantaa, Finland). The biocompatible photopolymer (Biomed Clear, a U.S. Pharmacopeia Class VI certified rigid material for biocompatible applications requiring long‐term skin or mucosal membrane contact^[^ ^55^ ^]^) for 3D printing was obtained from Formlabs (Sommerville, MA, USA), and the BaSO_4_ nanoparticles (100 nm, purity 99%) were ordered from Nanoshel (Dera Bassi, Punjab, India). Eudragit L100 was bought from Evonik Röhm GmbH (Darmstadt, Germany), dibutyalsebactate (DBS) and PBS were obtained from Sigma‐Aldrich (St. Louis, MO, USA), and 2‐propanol (IPA) was purchased from VWR International (Radnor, PA, USA). Fresh porcine small intestinal tissue from 50 to 55 kg Landrace × Yorkshire × Duroc pigs was donated by the Department of Experimental Medicine, University of Copenhagen (Copenhagen, Denmark), and it was cut into pieces with a length of ≈18 cm prior to storage at −20 °C until further use. The Male Sprague‐Dawley rats used were ordered from SCANBUR A/S (Karlslunde, Denmark).

### 3D Printing of Microcontainers

All of the microcontainer designs were drawn in a 3D software (SolidWorks, Dassault Systèmes SolidWorks Corporation, Waltham, MA, USA) used to generate corresponding computer‐aided design (CAD) files. Each of the CAD files was converted to G‐codes, which are computer numerical control commands, to execute the printing path and parameter. The HD‐DVD based 3D printer utilized a 405 nm wavelength laser to cure the photopolymer, and thereby fabricate the microcontainers, on 12.5 × 12.5 mm^2^ polished silicon substrates.^[^ ^27^ ^]^ The entire printing procedure consisted of printer alignment, printing process, cleaning, and post‐curing. For a single printing batch, the printer produced 100 microcontainers in a 10 × 10 array with a pitch of 900 µm between the microcontainers. The following parameter settings were used; a printing speed from 0.10 to 0.15 mm s^−1^, a laser intensity of 2.40 µW, and a photopolymer thickness of 25 µm. Each microcontainer design was a total of ten layers, each layer being 21 µm in height. The additive features of the pillar and fork designs consisted of six layers, each layer being 9 µm high. The printing process was operated at room temperature (20 °C) with a relative humidity below 30%. A compensation method was applied to tune the printing speed slightly for each of the microcontainer designs to maintain the exact laser exposure dosage during the printing process. In the cleaning process, the microcontainers were first immersed in IPA for 5 min and then continuously flushed with IPA until no residual photopolymer was left. As the solvent evaporated, the microcontainers were placed in a 405 nm wavelength chamber (Formlabs, Somerville, MA, USA) for 30 min of post‐curing.

The quality of empty, loaded, and coated 3D‐printed biocompatible microcontainers was investigated using a tabletop SEM (TM3030Plus, Hitachi High‐Technologies Europe, Krefeld, Germany) with a 45°‐tilted metallic holder carrying the specimen. The samples were imaged using an electron acceleration voltage of 5 keV and a backscattered electron detector in standard mode.

### In Vitro Proof‐of‐Concept Study

For an in vitro release study, the microcontainers were loaded with furosemide as previously reported.^[^ ^8^, ^56^ ^]^ Briefly explained, the furosemide was manually distributed on a microcontainer chip, and any excess drug between the microcontainers was subsequently removed using an air gun. After loading, an ultrasonic spray coater (ExactaCoat, Sono‐Tek, Milton, NY, USA) equipped with an accumist nozzle was applied to seal the furosemide‐loaded microcontainers with the pH‐sensitive polymer Eudragit L100. A solution of the polymer (1% w/v Eudragit and 5% w/w in relation to the polymer of DBS dissolved in IPA) was sprayed over a chip of drug‐loaded microcontainers. The specific settings applied for the spray coating process are described in detail in previous work.^[^ ^7^, ^10^ ^]^ In brief, the microcontainers were coated by 30 passages with a flow rate of 0.1 mL min^−1^ on a heating plate set to 40 °C. The release of furosemide was measured using a µDISS Profiler (Pion Inc., Billerica, MA, USA) as previously described in literature.^[^ ^16^ ^]^ To summarize, a microcontainer chip was fixed on top of a cylindrical magnetic stirring bar with double‐sided carbon tape, placed in a sample vial, and covered with 10 mL gastric buffer (hydrochloric acid, pH 2.4). Individually calibrated UV probes were immersed into the gastric buffer followed by initiation of the experiment. After 30 min, the release medium was changed to 10 mL intestinal buffer (PBS, pH 7.5). The release was measured at 37 °C with a stirring rate of 100 RPM and UV measurements (310–350 nm) were conducted every 10 s.

### Mucoadhesion Force Measurement

A wide‐range optical‐pickup‐unit force analyzer^[^ ^42^ ^]^ was utilized for ex vivo mucoadhesive force measurements. The force analyzer was equipped with a precise optical module and a cantilever force transducer to detect forces at the micronewton scale. As the cantilever structure transferred the applied force into deflection, the optical module sensed the cantilever deflection. The dimensions of the cantilever were 12 × 8.2 × 0.1 mm^3^ (width × length × thickness). A homemade instrument for alignment ensured precise placing of the microcontainers on the tip of the microprobe. Then, the microcontainers were mounted with UV cross‐linkable glue. Frozen porcine small intestinal tissue was thawed at room temperature for 30 min and sliced into small pieces of ≈20 mm in length. During the measuring process, the optical‐pickup‐unit force analyzer lifted the sample stage with tissue to approach the microcontainers with a constant speed of 0.078 mm s^−1^. While the microcontainer contacted the mucus layer, the platform lifted 80 µm to ensure complete contact between the microcontainer surface and the mucus. After 0.5 s contact time, the platform was withdrawn with the same constant speed of 0.078 mm s^−1^, detaching the microcontainers from the mucus layer. Simultaneously, the force analyzer recorded the cantilever deflection induced by the mucoadhesion force, plotting it into a force–displacement chart. The maximum point of the curve was defined as the peak mucoadhesion force. To compare the measured mucoadhesive forces, the microprobe was utilized as a blank sample to normalize the mucoadhesion between each of the test groups.

During the investigation of the microcontainer orientation, the force measurements of each design were first made with the order of blank, top side, bottom side, and blank at the exact location on small intestinal tissue (Figure S7A, Supporting Information). These measurements were repeated three times with a new piece of tissue for each round. After that, similar triplicate measurements were made with a swapped order of the top side and bottom side. For studying the additive features on the pillar and fork microcontainers, all measurements started and ended with a blank sample and with all six possible combinations in between (Figure S7B, Supporting Information).

The normalized peak mucoadhesion force was analyzed through statistical methods using data analysis software (RStudio, RStudio Inc., Boston, USA). A two‐way ANOVA test was implemented to validate the influence of top‐bottom orientation and overall design, respectively. In addition, a quantile‐quantile plot analysis showed the normal distribution, demonstrating the reliability of the data set. To further make a comparison of each design, Tukey's pairwise test was utilized (a link to an online available R script used for the data fit can be found in Supporting Information).

### 3D Printing of Radiopaque Microcontainers

To fabricate 3D‐printed microcontainers that were traceable with X‐ray imaging, BaSO_4_ nanoparticles (37.9 w/v%) were mechanically mixed into the 3D printing resin. The shape, chemical composition, and homogeneity of the 3D‐printed radiopaque microcontainers were investigated using SEM, EDX, and µCT scanning, respectively. The SEM investigation was conducted as previously described but EDX (80 mm^2^ X‐Max silicon drift detector, Oxford Instruments, Abingdon, Oxfordshire, United Kingdom) analysis was included. For µCT scanning (ZEISS XRadia 410 Versa, ZEISS, Pleasanton, CA, USA) of the 3D‐printed radiopaque microcontainers, the distance between the X‐ray probe and the sample was set to obtain a voxel size, which corresponds to the spatial scan resolution, of 4.468 µm. X‐rays were generated using a voltage of 60 kV and a power of 10 W (current of 0.17 mA). The respective 3D visualizations for all samples were created from single planar scans using 3202 projections with one frame per projection and an exposure time of 2 s. The final scan time was 3 h and 3 min. The following tomographic reconstructions were made in the software provided with the µCT scanner system (Scout‐and‐Scan Control System Reconstructor, ZEISS, Pleasanton, CA, USA) using a Feldkamp, Davis, and Kress algorithm that is a filtered back‐projection algorithm.^[^ ^57^ ^]^ The reconstructed data were processed and investigated using a 3D visualization and analysis software (Avizo, Thermo Fisher Scientific Inc., Waltham, MA, USA).

### In Vivo Rat Study

All animal care, housing, and procedures were performed at the Bio Facility at the Technical University of Denmark. The study was approved by The Danish Animal Experiments Inspectorate under the license 2020–15–0201–00610 and conducted in compliance with the Danish laws regulating experiments on animals and the EC Directive 2010/63/EU. For this study, male Sprague‐Dawley rats with a weight of 288–320 g were used. The rats were acclimatized for one week prior to the study at a room temperature and humidity of 22 °C and 55%, respectively, and with an alternating 12/12 h light/dark cycle. During the entire acclimatization period, the rats had free access to standard food pellets and water. In total, 24 rats were used, and a detailed experimental design of the in vivo rat study was made (Table S2, Supporting Information). The rats were fasted 16–18 h before gelatin capsules filled with 50 microcontainers, having either neutral, pillar, or arrow design, were administered by oral gavage. This was done using a device with a stick dosing mechanism, which ensured the gelatin capsules to be expelled from the device without introducing air or water into the stomach of the rats. After oral administration, the rats were given free access to water throughout the study. The rats were euthanized in groups 0.5–3 h post‐administration by gassing for 2–3 min with a 60% carbon dioxide in 40% oxygen mixture directly followed by decapitation. After euthanasia, all rat GI tracts from the stomach to the terminal end of rectum were removed. The stomachs and small intestines were placed in large Petri dishes, whereas the ceca and colons were placed in small Petri dishes. The GI tracts were frozen and stored at −18 °C until later planar X‐ray imaging was performed. After planar X‐ray imaging of the entire GI tracts, small intestinal pieces containing microcontainers were cut from one of the two 0.5 h samples in each group of microcontainer design. These three small intestinal pieces were first placed hanging inside Falcon using needles for CT scanning and then, they were opened using a scissor prior to investigation with CryoSEM.

### Planar X‐Ray Imaging and Quantification of Microcontainers

Planar X‐ray imaging of the entire removed and frozen GI tracts for quantitative tracking of the microcontainers was carried out using a CT scanner (Nikon XT H 225, Nikon Metrology, Tokyo, Japan). The distance between the X‐ray probe and the samples was set to get at a magnification of 2.5, and X‐rays were generated using a voltage of 70 kV and a power of 30 W (current of 0.43 mA). Acquisition of planar X‐ray images, including a background signal for shading correction, was done using eight frames with an exposure time of 1 s for each. Shading corrections and subsequent manual quantification of microcontainers in predefined different sections of the entire GI tracts (stomach, proximal and distal small intestine, cecum, and colon) were performed by two independent persons using an image processing software (ImageJ, freeware).

### Graphical Representation of Gastrointestinal Retention and Transit Time

The raw counts obtained, by two independent persons during the manual quantification of microcontainers in the different GI sections, were almost identical and therefore averaged in order to minimize any potential counting errors. Furthermore, these averaged raw counts were normalized to the total quantity of microcontainers found for each of the individual rats to account for microcontainers not found and counted. By doing so, the nonidentified microcontainers were evenly included according to the number of microcontainers actually found in each of the GI sections, which made overall data comparison easier. The final averaged and normalized counts for each microcontainer design in the different sections of the GI tract were plotted and fitted in a data analysis software (Python, Python Software Foundation, Wilmington, DE, USA) as Gaussian processes over time with indications of the fitted variances (detailed information on this, including a link to an online available Python script used for the data fit and plotting, can be found in Supporting Information). For this, the microcontainer counts in the stomach section at 0 h (immediately after oral dosing of the microcontainers) was set to 100%, whereas the counts at 0 h for all of the remaining GI sections was set to 0. In order to compare potential differences in retention and transit time between the three designs, especially relevant in the small intestinal sections (proximal and distal), the fits of these data were overlayed accordingly in single graphs.

### CT Scanning and CryoSEM of Small Intestinal Pieces

For visualization of the microcontainers in 3D inside the small intestine, the three previously mentioned frozen small intestinal pieces from the 0.5 h samples were CT‐scanned. The distance between the X‐ray probe and the sample was fixed to get a magnification of 20 for all scans in order to keep the voxel size, corresponding to the spatial resolution, constant at 19.962 µm. X‐rays were generated using a voltage of 70 kV and a power of 25 W (current of 0.36 mA). Each of the 3D visualizations were created from single planar scans using 1572 projections with two frames per projection and an exposure time of 1 s, which gave a final scan time of 53 min. The following tomographic reconstructions were made using a filtered back‐projection algorithm, Feldkamp, Davis, and Kress algorithm,^[^ ^57^ ^]^ in the software provided with the CT scanner system (CT Pro 3D, Nikon Metrology, Tokyo, Japan). Finally, a 3D visualization and analysis software (same as previous) was used to process and investigate the CT scan data.

In addition, parts of the three frozen small intestinal pieces were subsequently investigated by a CryoSEM. The CryoSEM installs a Quorum PP2000 Cryo‐System (Quorum Technologies, East Sussex, United Kingdom) on a Quanta FEG 200 ESEM (Field Electron and Ion Company, Oregon, United States). The sample was observed using an electron acceleration voltage of 10 keV, and a secondary electron detector at −180 °C.

## Conflict of Interest

The authors declare no conflict of interest.

## Author Contributions

T.‐J.C. and R.B.K. contributed equally to this work. T.‐J.C., R.B.K., J.F.C., and L.H.N. contributed to conceptualization and methodology. T.‐J.C. designed and fabricated the 3D printed microdevices, and implemented ex vivo mucoadhesion force measurement. R.B.K. implemented in vivo rat study and quantitative gastrointestinal tracking for retention and transit time. J.F.C. contributed to in vitro proof‐of‐concept for controlled oral drug delivery. E.M.V. contributed to the incorporation of BaSO_4_ into photopolymer for 3D printing. T.S.A. implemented the statistical analysis of ex vivo mucoadhesion force measurement and quantitative gastrointestinal tracking. T.‐J.C., R.B.K., and J.F.C. contributed to the visualization and the original draft writing with the assistance of all authors. E.‐T.H., K.Z., L.H.N., and A.B. supervised the project and reviewed the writing. A.B. contributed to the funding acquisition and resources.

## Supporting information

Supporting Information

Supplemental Movie 1

Supplemental Movie 2

Supplemental Movie 3

## Data Availability

The data that support the findings of this study are available in the supplementary material of this article.
