# Supplementary material for: 3D‐Printed Radiopaque Microdevices with Enhanced Mucoadhesive Geometry for Oral Drug Delivery
Source: Adv Healthc Mater. 2022 Dec 4;12(4):2201897. doi: 10.1002/adhm.202201897 (PMC11468800; doi:10.1002/adhm.202201897)
Supplement: Supplementary file 1 — Supporting Information [file ADHM-12-2201897-s004.pdf]

Supporting Information for

3D-printed radiopaque microdevices with enhanced mucoadhesive geometry for oral drug delivery

This PDF file includes:

Supplementary text (a single combined section)
Figure S1 to S7
Table S1 to S2

Other Supporting Information (available online) includes the following:

Movies S1 to S3

Supplementary text (regarding Figure 3D, S5 and 6)

An R and Python script used, respectively, to compute the statistics for Figure 3D and for data fit and plotting in Figure S5 and 6 are available at:
https://doi.org/10.11583/DTU.c.6310968

The data fitting in Figure S5 and 6 was made using a Gaussian process with a squared exponential kernel and a Poisson likelihood. The inference was carried out using the Laplace approximation, so it is important to remark that the variances are crude approximations of the process variance. The exponential kernel has two hyperparameters: 1) the length-scale, which controls the smoothness of the fit, and 2) the variance, which controls how strong an emphasis the process will have on the observations. In order to ensure that the fifteen different fits would have comparable hyperparameters, they were regularized using a Gamma prior distribution. The length-scale follows a Gamma distribution with the mean value of 1 and variance of 0.01, and the variance follows a Gamma distribution of mean value 0.5 and variance 0.01.

Supplementary figures and tables

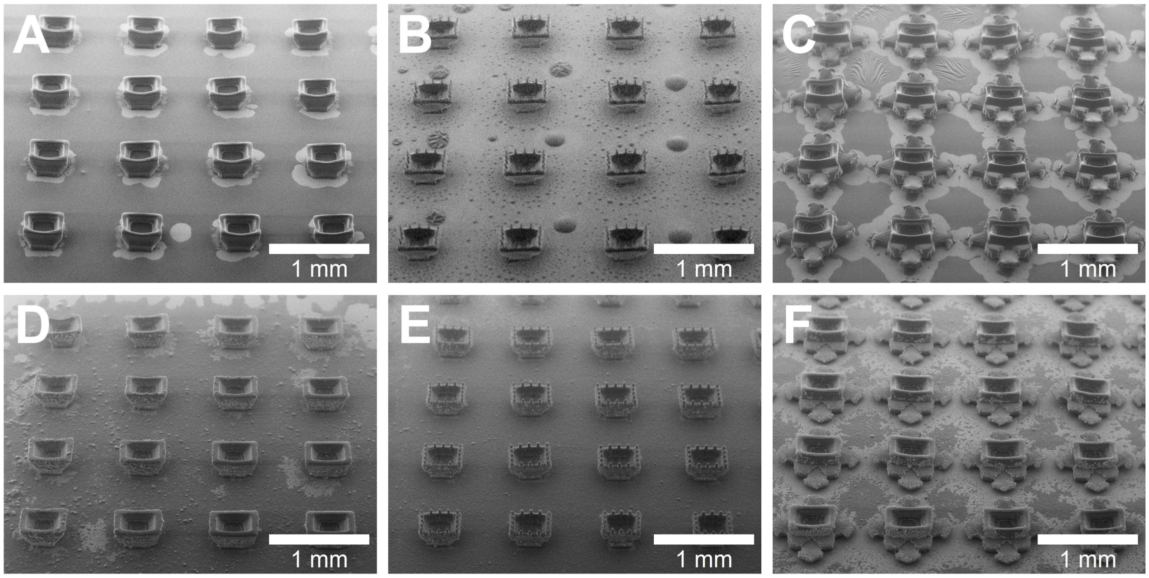

Figure S1. Mass-production of 3D printed microcontainers. SEM images showing the homogeneity of mass-produced 3D printed microcontainers A-C) without and D-F) with $BaSO_4$, respectively, having neutral, pillar and arrow design (supplement to Figure 2D-F and Figure 4A-C, respectively).

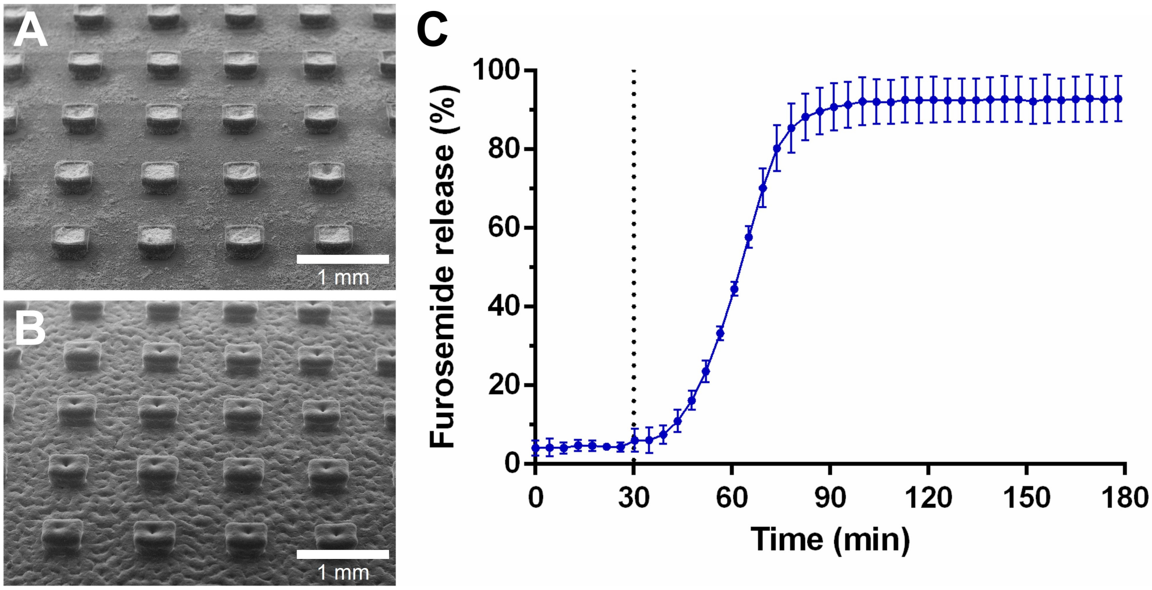

Figure S2. Proof-of-concept for targeted oral drug delivery. SEM images of 3D printed microcontainers A) loaded with furosemide and B) coated with Eudragit® L100. C) *In vitro* drug release of furosemide from the loaded and coated microcontainers in gastric pH during the first 30 min (pH 2.4) followed by an intestinal step (pH 7.5). Mean ± SD, n = 4.

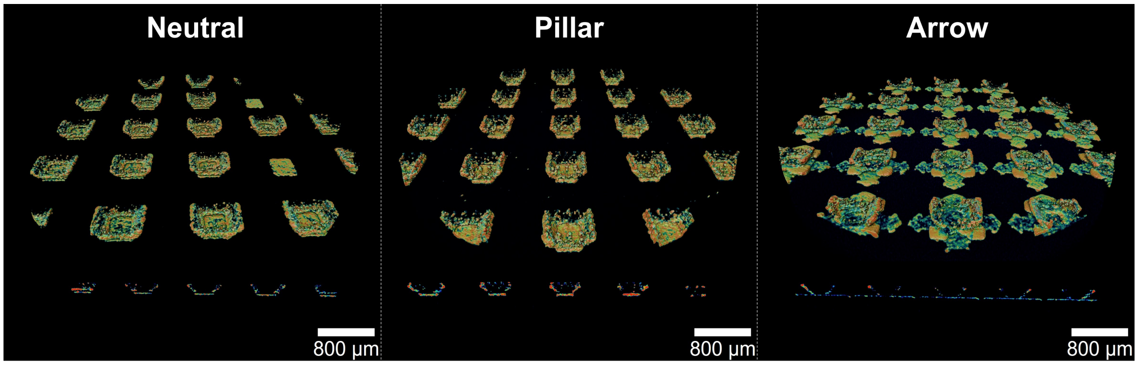

Figure S3. µCT scanning of microcontainers. µCT scanning showing the homogeneity of $BaSO_4$ nanoparticles in the 3D printed radiopaque microcontainers (supplement to Figure 5B).

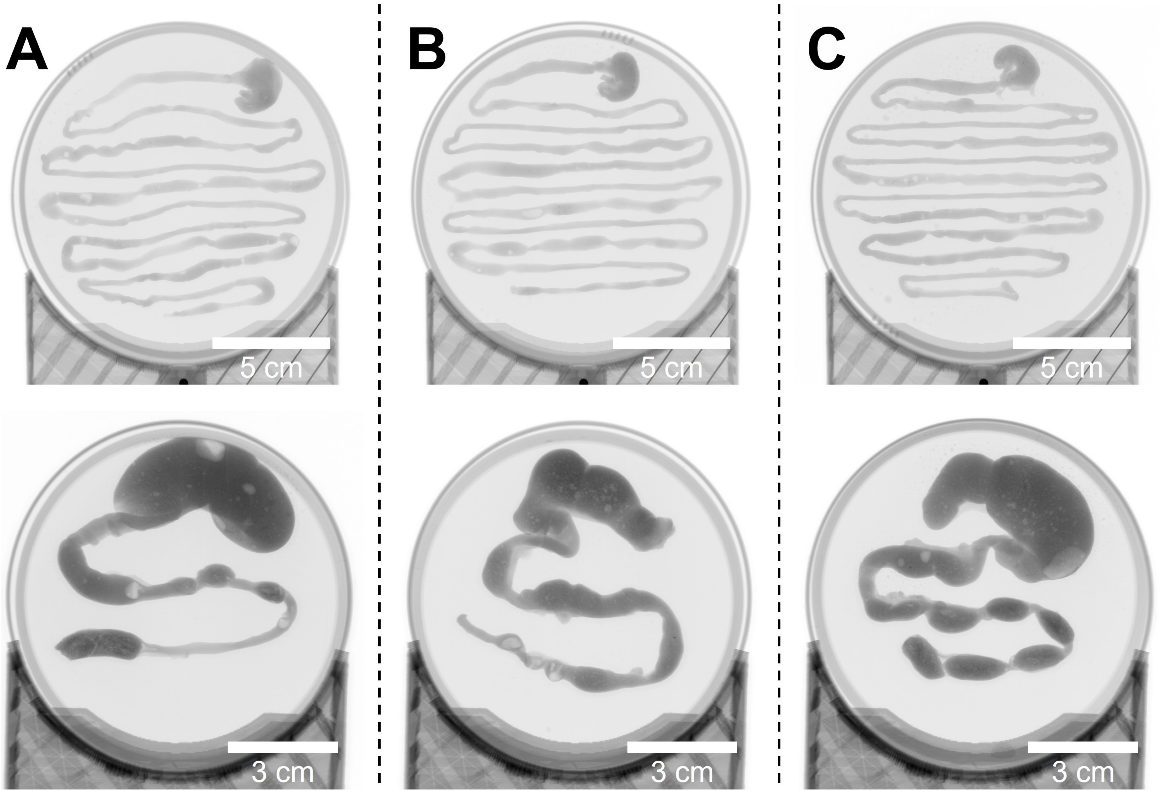

Figure S4. Representative GI tracts. Planar x-ray images of three representative removed GI tracts at 0.5 h. A) neutral design, B) pillar design, and C) arrow design.

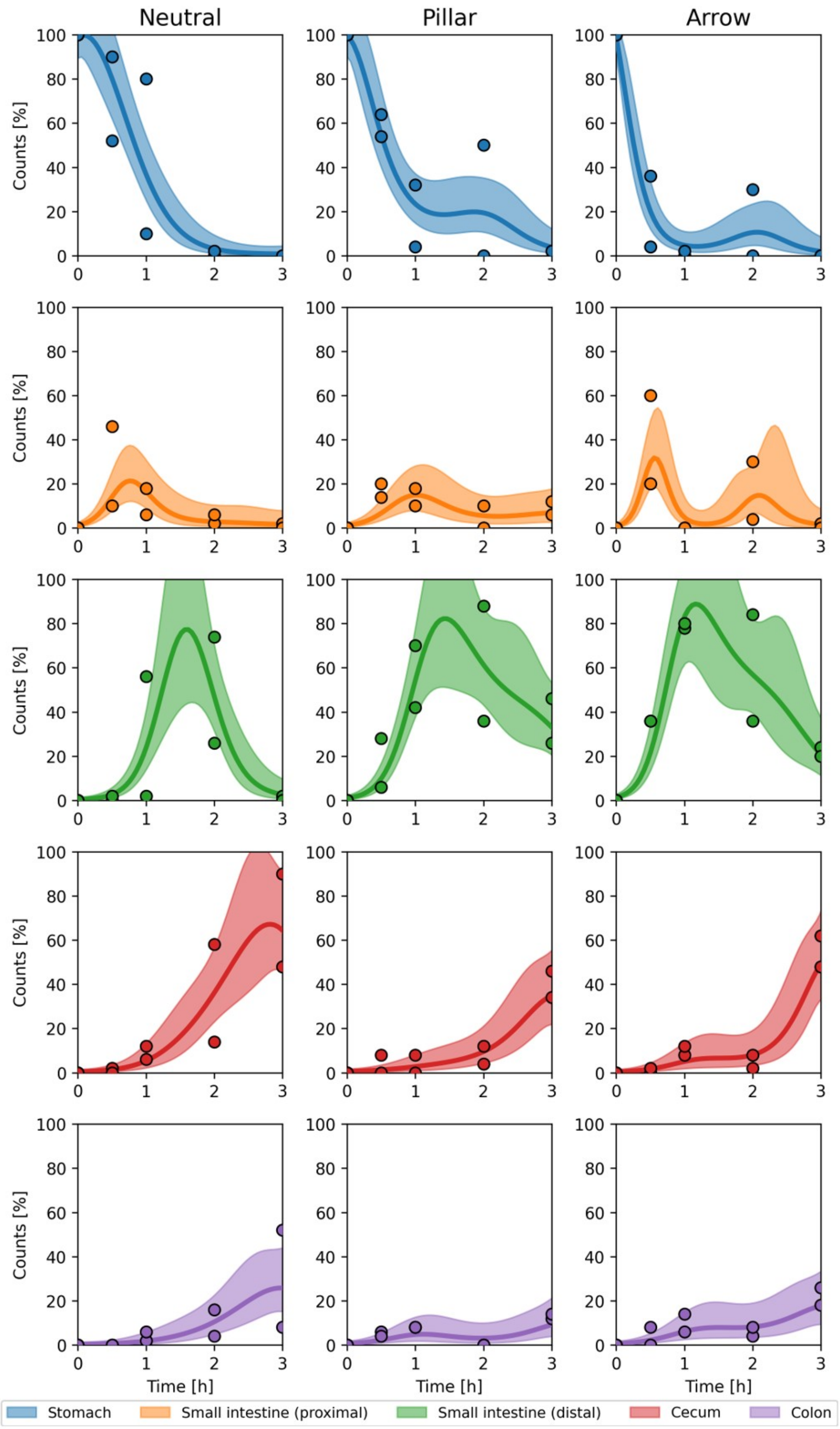
Neutral
Pillar
Arrow
Counts [%]
Time [h]
Stomach
Small intestine (proximal)
Small intestine (distal)
Cecum
Colon

Figure S5. Plots of averaged and normalized data for GI retention and transit time. Graphs showing all data points obtained for GI retention and transit time for each design of microcontainers (Table S1) with the fitted curves and indications of the fitted variances.

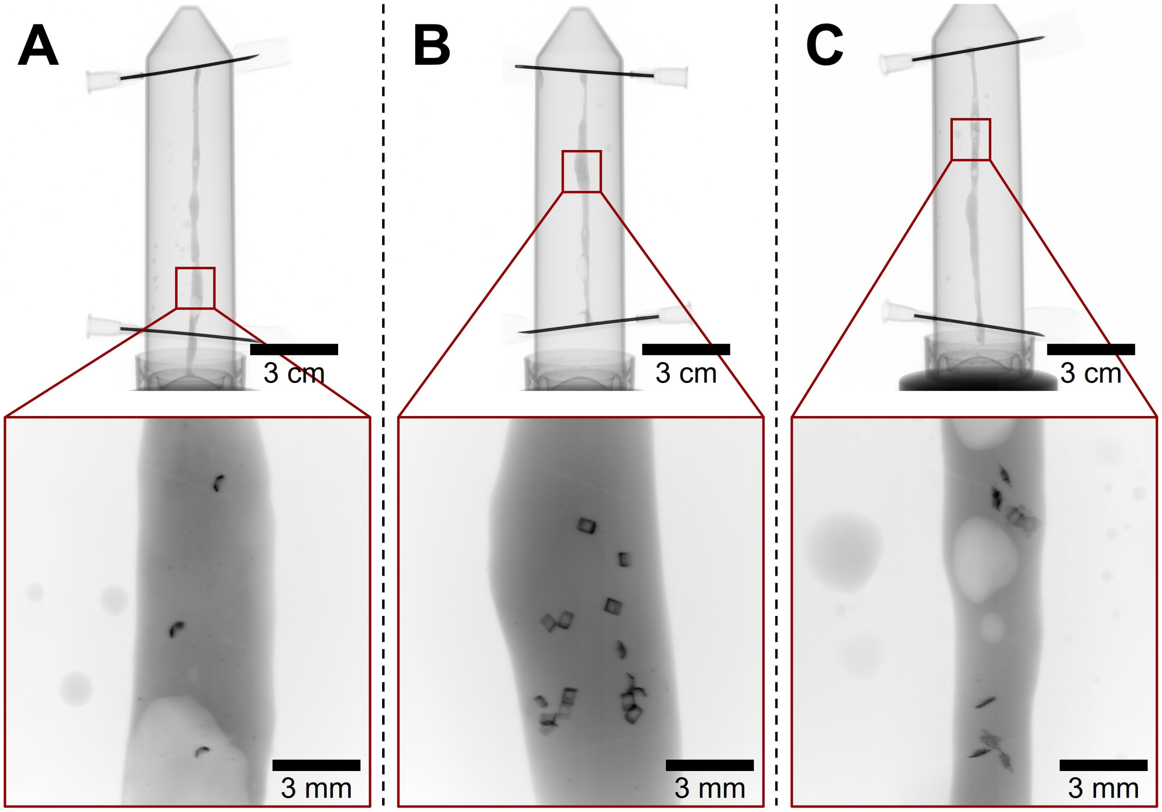

Figure S6. Samples for CT scanning and CryoSEM. Planar x-ray images of the three samples used for CT scanning and CryoSEM. A) neutral design, B) pillar design, and C) arrow design.

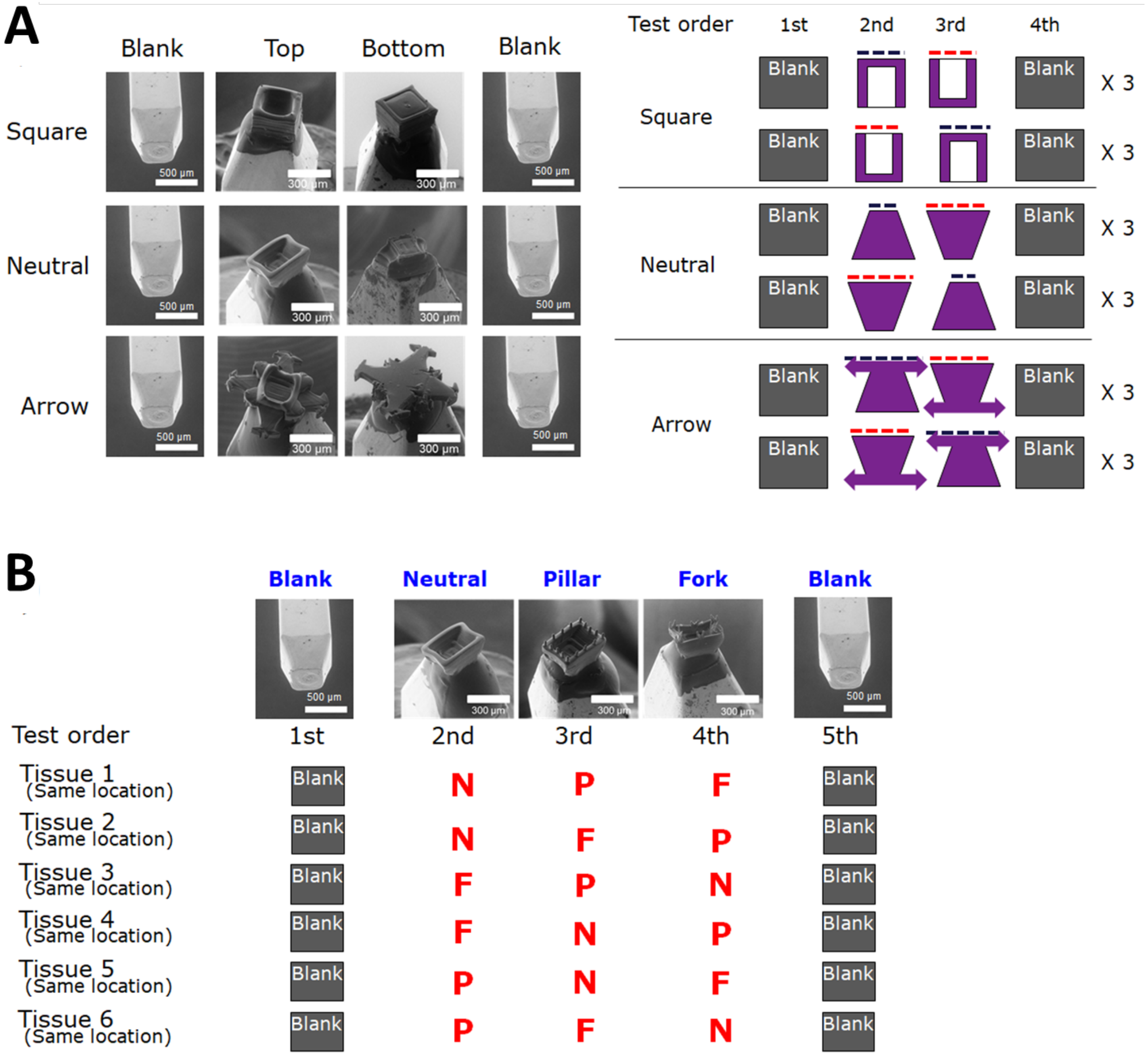

Figure S7. Experimental designs for *ex vivo* mucoadhesion measurements. Test orders used during the force measurements of A) top and bottom of square, neutral and arrow microcontainers (two sets of triplicates), respectively, and B) top of neutral, pillar and fork microcontainers (all six possible combinations).

Table S1. Averaged and normalized data for GI retention and transit time. The raw counts obtained, by two independent persons, were averaged and then normalized to the total amount of microcontainers found for each of the individual rats in order to minimize any potential counting errors and to make overall comparison easier, respectively. All data has been plotted and fitted for easier comparison (Figure S5).

	Time [h]	Stomach [%]	Small intestine (proximal) [%]	Small intestine (distal) [%]	Cecum [%]	Colon [%]
**Neutral**	0.5	51.85	45.68	0.00	2.47	0.00
		89.02	9.76	1.22	0.00	0.00
	1	10.58	17.31	56.73	12.50	2.88
		79.80	6.06	2.02	6.06	6.06
	2	0.00	1.02	25.51	58.16	15.31
		1.80	5.41	74.77	13.51	4.50
	3	0.00	1.19	1.19	89.29	8.33
		0.00	0.00	0.00	47.00	53.00
**Pillar**	0.5	53.26	13.04	27.17	0.00	6.52
		63.24	19.12	5.88	7.35	4.41
	1	31.25	17.86	41.96	0.00	8.93
		4.12	10.31	70.10	7.22	8.25
	2	0.00	0.00	88.24	11.76	0.00
		50.00	9.62	35.58	4.81	0.00
	3	2.30	5.75	45.98	33.33	12.64
		1.90	11.43	26.67	45.71	14.29
**Arrow**	0.5	4.00	59.00	37.00	0.00	0.00
		35.11	20.21	35.11	2.13	7.45
	1	0.00	0.00	78.95	7.37	13.68
		2.33	0.00	80.23	11.63	5.81
	2	29.21	29.21	35.96	2.25	3.37
		0.00	3.00	83.00	7.00	7.00
	3	0.00	1.92	23.08	48.08	26.92
		0.00	0.00	19.78	61.54	18.68

Table S2. Experimental design of *in vivo* rat study and following examination. 24 rats in three different groups were totally used for the *in vivo* rat study. Indications "+" and "–" means yes and no, respectively, in relation to the following examination.

	**Neutral**				**Pillar**				**Arrow**			
Euthanasia [h]	0.5	1	2	3	0.5	1	2	3	0.5	1	2	3
Number of rats	2	2	2	2	2	2	2	2	2	2	2	2
Removal of GI tract	+	+	+	+	+	+	+	+	+	+	+	+
Planar x-ray imaging of GI tract and manual counting	+	+	+	+	+	+	+	+	+	+	+	+
CT scanning and CryoSEM of small intestinal piece	+	–	–	– –	+	–	–	– –	+	–	–	– –
